# A Biomass Based Photonic Crystal Hydrogel Made of *Bletilla striata* Polysaccharide

**DOI:** 10.3390/bios12100841

**Published:** 2022-10-08

**Authors:** Bo Sun, Wenxin Zhang, Yangyang Liu, Min Xue, Lili Qiu, Zihui Meng

**Affiliations:** School of Chemistry and Chemical Engineering, Beijing Institute of Technology, Beijing 100081, China

**Keywords:** photonic crystals, *Bletilla striata* polysaccharide, hydrogel, semi-interpenetrating network, sensor

## Abstract

*Bletilla striata* is an herb with a good medicinal value whose main active ingredient is *Bletilla striata* polysaccharide (BSP) in the tuber of *Bletilla striata*. In this study, a polysaccharide-based semi-interpenetrating network hydrogel was constructed by introducing BSP into polyacrylamide (PAM) hydrogel. The introduction of the BSP chain no only maintains the excellent mechanical properties of PAM, but also endows it with good biocompatibility. By implanting the colloidal crystal array into the above hydrogels, we obtained a novel biomass-based photonic crystal with good stimulus responsiveness that is sensitive to volatile organic compounds (VOCs), especially alcohol vapor. In addition, due to the scavenging ability of BSP to hydroxyl radicals, the photonic crystal hydrogel also has a good response to hydrogen peroxide ($H_{2}O_{2}$).

## 1. Introduction

Photonic crystals (PhCs) have nanoscale periodic structures [1], and the existence of photonic band gaps gives them unique optical properties. Since Yablonovitc [2] and John [3] independently proposed the concept of photonic crystals in 1987, PhCs have been applied in many areas of sensor technology, such as temperature [4], humidity [5], solvent [6], mechanical force [7,8], glucose [9], pH [10], and metal ions [11,12,13].

The semi-interpenetrating network (Semi-IPN) hydrogel is one or more linear macromolecules penetrating through the middle of the polymer, which means that among the two polymers that make up the interpenetrating network (IPN), only one polymer is an interpenetrating network. The other polymer is linear non-crosslinked. The linear macromolecular chain and the network structure of the polymer are not chemically combined, but only physically penetrated [14]. Some natural polysaccharides and their derivatives are macromolecular chains in natural polymers such as cellulose [15], alginate [16], konjac glucomannan [17], silk fibroin [18], gelatin [19], and chitosan [20]. Zhang et al. [21] used Kappa-carrageenan to form a semi-interpenetrating structure with a C2-symmetric benzene-based supramolecular gelator to effectively improve the surface wettability of the gel. Min et al. [22] hybridized alginate with polyvinyl alcohol (PVA) to form a semi-interpenetrating structure, which exhibited excellent stability. Wang et al. [23] introduced chitosan and gelatin, which greatly improved the copper ion adsorption capacity of polyacrylic acid hydrogels. The introduction of natural polysaccharides makes the semi-interpenetrating network hydrogels have good biocompatibility and degradability, while maintaining the original physicochemical properties of polymers, and is widely used in biomedical materials.

In recent decades, scientists have shown great interest in stimuli-responsive hydrogels. These smart hydrogels can exhibit stimuli-responsive changes in their volume and structure, enabling various applications [24]. Optical sensing based on PhCs has several benefits such as low cost, accuracy, rapid response, and consistency of results [25,26,27,28,29,30]. As far as the PC-based biosensor sensitivity is concerned, many papers have mainly focused on the means of enhancing the sensitivity by many techniques [31,32,33].

*Bletilla striata* is a perennial herb with good medicinal value [34], and the main active ingredient with multiple biological functions is *Bletilla striata* polysaccharide (BSP) [35]. The main components of BSP are similar to konjac glucomannan, and its backbone is mainly 1,2- or 1,4-linked mannose residues and 1,4-linked glucose residues. The molar ratio of branched polysaccharides consisting of mannose and glucose is 2.4:1 [36]. Pharmacological studies have shown that BSP has various biological activities such as antioxidant [37], anti-inflammatory [38], antibacterial [39], and antitumor [40]. Due to its good biocompatibility, its own non-toxicity, and easy modification, BSP is widely used in medicine [41] and cosmetic industries [42], and has broad application prospects in the fields of pharmaceutical raw materials, biomedical materials, and pharmaceutical excipients.

Herein, we proposed a simple preparation method for a novel BSP-based Semi-IPN hydrogel. The introduction of BSP not only maintained the excellent mechanical properties of PAM hydrogels, but also gave the hydrogels good biocompatibility. By combining this hydrogel with PhCs, we obtained a BSP-based biomass PhCs hydrogel with good responsiveness to chemical stimuli from VOCs (especially ethanol). In addition, due to the scavenging effect of BSP on hydroxyl radicals, the hydrogel also has good sensing performance for $H_{2}O_{2}$, which has a good application prospect.

## 2. Materials and Methods

Methacrylic acid (MAA), potassium peroxydisulfate (KPS), *N*,*N*’-Methylene bisacrylamide (MBA), and Phenylglyoxal diethyl acetal (DEAP) were purchased from J&K Scientific (Beijing, China). Bletilla striata polysaccharide (BSP) was obtained from Xian Yunhe Bio-Technology Co., Ltd. (Xi’an, China). Acrylamide (AM) was purchased from TCI (Shanghai, China). Methyl alcohol, ethanol, acetonitrile, acetone, and other affiliated chemicals were all obtained from Beijing Chemical Industries (Beijing, China). All reagents used in the experiment were analytical quality unless clarified specifically. Ordinary glass slides (76.2 mm × 25.4 mm × 1 mm) were purchased from Sail Brand (Shanghai, China). Cover glasses were purchased from Citotest Labware Manufacturing Co., Ltd. (Nantong, China).

### 2.1. Formation of 3D Photonic Crystal

The basic alumina (200–300 mesh) was used to remove the polymerization inhibitor in the MMA solution by column chromatography; 290 mL of water was added to a 500 mL four-neck flask equipped with a thermometer, condensed water, and mechanical stirring, with the stirring speed kept at 250 rpm, and nitrogen passed for 20 min. The temperature was raised to 75 °C, and a certain amount (10 mL–25 mL) of monomer MMA was added, followed by raising the temperature to 80 °C, and 15 mL initiator KPS aqueous solution at a concentration of 0.04 g/mL was added after the temperature was stabilized for 10 min. The reaction was refluxed for 45 min, and PMMA microspheres were prepared. After the polymerization reaction, the mixture was centrifuged and washed three times with water to remove the unreacted monomer and initiator. The slides were hydrophilized with a plasma cleaner. The concentration of the PMMA solution was then adjusted to 2 mg/mL. Finally, three-dimensional photonic crystals were prepared by the vertical sedimentation method. The slides were placed vertically in the PMMA solution which was allowed to evaporate at RH 50% and 30 °C.

### 2.2. Formation of Semi-IPN Photonic Crystal Hydrogel

In total, 0.2 g AM, BSP (5−20% to AM), and 0.002 g MBA were dissolved in 2 mL water, then 0.2 mL DEAP of 0.01 g/mL was added via ultrasonic mixing to prepare a prepolymer solution. The three-dimensional photonic crystal array was stacked between two glass slides of the same size, and separated by a certain thickness of white tape to control the thickness. A certain amount of the above gel prepolymer solution was slowly injected into the above “sandwich” structure, and under the action of capillary force, the gaps between the glass slides were completely filled with the prepolymer solution. Then the solution was photopolymerized under a UV cross-linker (365 nm) for 10 min. Finally, the gel film was swollen and peeled off the glass slide automatically by soaking it in pure water, and the BSP-PAM photonic crystal hydrogel was obtained.

### 2.3. Characterization

The microscopic morphology and size of BSP-PAM photonic crystal hydrogel were characterized with a Hitachi S-4800 field emission scanning electron microscope under 10 kV accelerating voltage. Fourier-transform infrared spectroscopy (FTIR) spectra were obtained using a Thermo Nicolet iS5 spectrometer. The FTIR spectrum was recorded in attenuated total reflection mode with a spectral range of 400–4000 cm^−1^. The structural color of the photonic crystal and the actual picture of the hydrogel were recorded by the camera. PMMA microspheres were mainly prepared by an RW20 digital agitator, an IKA C-MAGHS7 temperature controller, and an Anke TDL-60B centrifuge; PMMA three-dimensional photonic crystal gel film was mainly prepared by a Xinzhi SCIENT203-II purple diplomatic instrument (Ningbo, China) and a Shanghai Shengyan SCQ-5201 ultrasonic cleaning instrument (Shanghai, China). The reflection peaks of photonic crystals and hydrogels were detected by optical fiber spectrometer (A-2048TEC, Avantes, Beijing, China).

## 3. Results

### 3.1. Characteristics of BSP-PAM Semi-IPN PhCs Hydrogel

**Figure 1:**
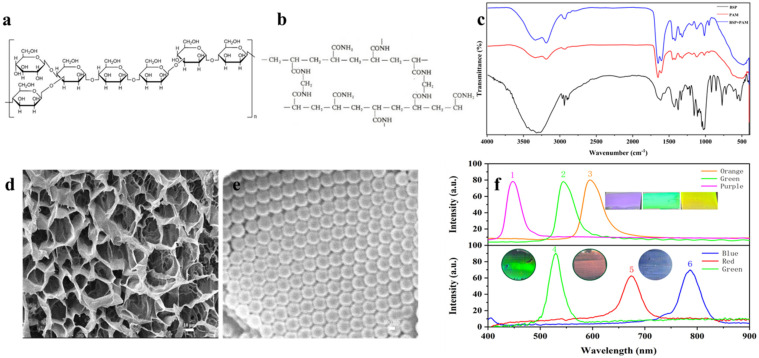
(**a**) Chemical structure of BSP. (**b**) Chemical network structure of polyacrylamide. (**c**) FT-IR spectra of BSP, PAM, and BSP-PAM. (**d**) SEM image of the network structure of BSP-PAM hydrogel. (**e**) SEM image of the BSP-PAM photonic crystal hydrogel. (**f**) Reflectance spectra of 3D photonic crystal arrays (1, 2, 3) and photonic crystal hydrogels (4, 5, 6); the inserted photos are the corresponding structural colors, respectively.

BSP is rich in hydroxyl groups (Figure 1a). By ultrasonically mixing BSP with monomer AM, cross-linker MBA, and photoinitiator DEAP as a pre-polymerization solution, under UV light irradiation, the chain-like BSP crosslinked inside the three-dimensional mesh structure of PAM (Figure 1d). At the same time, the hydroxyl groups on BSP formed hydrogen bonds with the amino groups on AM, and we obtained BSP-PAM Semi-IPN hydrogels. The infrared spectrum (Figure 1c) shows that the absorption peaks of BSP-PAM are significantly enhanced compared to AM (especially the peak at 1017 cm^−1^), the absorption peak at 500 cm^−1^ is red-shifted, and the absorption peak at 3317 cm^−1^ is also red-shifted in relation to BSP, which is caused by intermolecular hydrogen bonding, demonstrating the formation of a semi-interpenetrating structure. By infiltrating the prepolymer solution into the photonic crystal array via capillary force, the photonic crystal hydrogel with bright structural color can be obtained under ultraviolet light. It could be observed from scanning electron microscopy (SEM) (Figure 1e and Figure S2) that the periodic close-packed structure of the 3D PMMA array was not destroyed and completely embedded in the hydrogel. PMMA arrays with three particle sizes and their corresponding photonic crystal hydrogels were prepared using the methods in Section 2.1 and Section 2.2, respectively, and their reflection peak wavelengths were measured by a fiber optic spectrometer (Figure 1f). Compared with the photonic crystal array, the reflection peaks of the photonic crystal hydrogel have different degrees of redshift, which is formed by the increase of the lattice spacing of the PMMA microspheres due to the swelling effect of the hydrogel. The digital images are photographs of the photonic crystal array and gel corresponding to respective reflection peaks; the three images on top are the PMMA arrays and the three images below are the corresponding photonic crystal hydrogels. All of the photonic crystal hydrogels show bright structural colors and strong reflection peaks, and the third photonic crystal hydrogel shows a blue-violet color with secondary diffraction due to the reflection peaks beyond the visible range.

### 3.2. Response of BSP-PAM Semi-IPN PhCs Hydrogel to Ethanol

**Figure 2:**
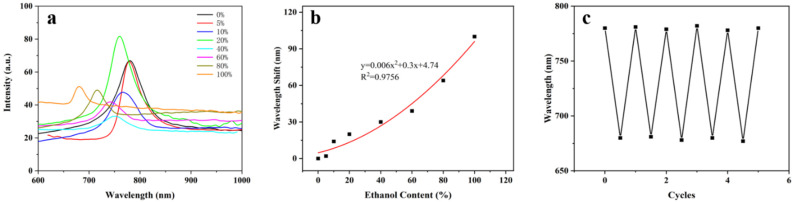
(**a**) Response of photonic crystal hydrogel to ethanol solution. (**b**) The relationship between redshift of reflection peak of the photonic crystal hydrogel and the concentration of ethanol. (**c**) Reproducible detection of photonic crystal hydrogels.

The stimuli-response of the semi-IPN hydrogels towards changes in ethanol concentration are presented in sensitive changes in reflectance wavelength. The BSP-PAM (15%) PhCs hydrogels were placed in different concentrations of ethanol solutions (5%, 10%, 20%, 40%, 60%, 80%, and 100%). With the increase of the ethanol content, the blueshift of the reflection peaks of the BSP-PAM PhCs hydrogels gradually increased (Figure 2a), and the ethanol concentration showed a good correlation with the reflection peak wavelength (Figure 2b). BSP is a hydrophilic polymer which can be well dissolved in water but insoluble in ethanol. In the ethanol solution, the BSP-PAM photonic crystal hydrogel shrunk by dehydration, resulting in the reduction of the distance between PMMA microspheres and the reduction of the lattice constant, which led to the blue shift of the reflection spectrum of the photonic crystal. After five rounds of repeatability testing on the photonic crystal hydrogel (Figure 2c), it still maintained good sensing performance, indicating excellent stability and repeatability.

### 3.3. Response of BSP-PAM Semi-IPN PhCs Hydrogel to VOCs

**Figure 3:**
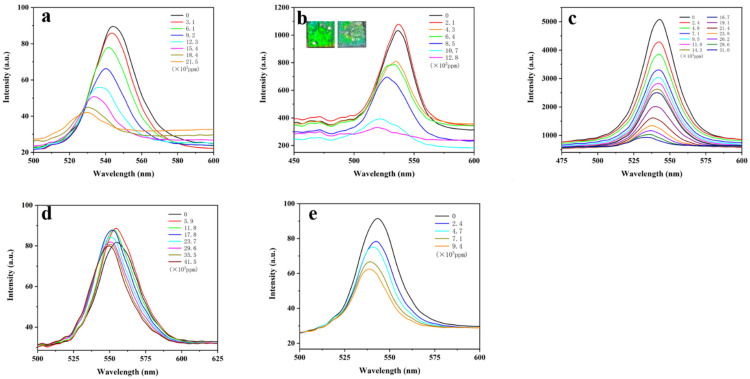
Response of photonic crystal hydrogel to methanol (**a**), ethanol (**b**), acetonitrile (**c**), acetone (**d**), and toluene (**e**) vapors.

**Figure 4:**
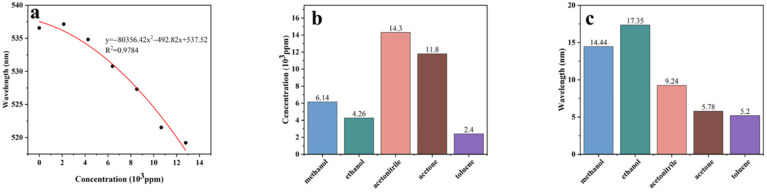
(**a**) The relationship between reflection peak of photonic crystal hydrogel and ethanol vapor concentration. (**b**) The lowest detection limit of the photonic crystal hydrogel for five VOCs. (**c**) The maximum redshift of the photonic crystal hydrogels for five VOCs.

Volatile organic compounds (VOCs) can cause serious harm to humans and the environment, so it is important to monitor VOC concentrations in the environment. A sealed device was constructed for the detection of VOCs, and the organic solvents were injected through a needle to generate vapor. The vapor concentration was calculated according to the following formula (Equation (1)):

(1) $$C\left(ppm\right)=\frac{\left(\frac{\rho V}{M}\right)\times 10^{6}}{\frac{V_{0}}{22.4}}=\frac{22.4\rho V}{MV_{0}}\times 10^{6}$$

where ρ is the density of the solvent (g/mL), *V* is the volume of the injected solvent (mL), $V_{0}$ is the volume of the container (L), and M is the relative molecular mass of the solvent (g/mol). BSP-PAM (15%) is sensitive to VOCs. We detected five VOCs: methanol, ethanol, acetonitrile, acetone, and toluene (Figure 3). With the increase of gas concentration in the device, the reflection peaks of BSP-PAM PhCs hydrogel gradually blue shifted to different degrees, which is due to the increase in the adsorption capacity of BSP-PAM PhCs hydrogel to vapors. Alcohol vapors caused the hydrogel to shrink by dehydration, acetone and toluene vapors made PMMA partially dissolve, and the lattice spacing decreasing. BSP-PAM PhCs hydrogel has the highest sensitivity to alcohol vapors (Figure 4), where the maximum redshift (λmax) of ethanol gas can reach 17.35 nm with a detection threshold of 4.26 × 10^3^ ppm, and λmax is well correlated with the concentration of ethanol vapor. PhCs hydrogel surroundings changed from green to cyan after detection of ethanol gas (inset in Figure 3b).

### 3.4. Response of BSP-PAM Semi-IPN PhCs Hydrogel to $H_{2}O_{2}$

**Figure 5:**
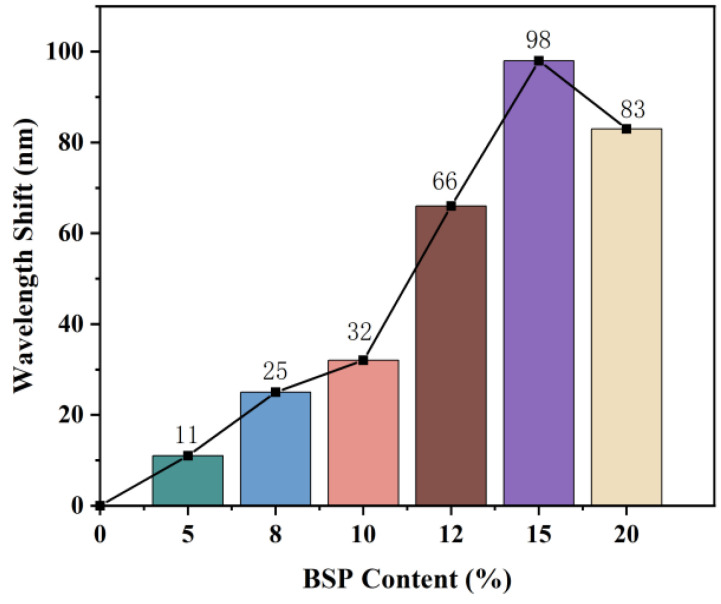
The maximum redshift of photonic crystal hydrogel with different BSP contents in 30% $H_{2}O_{2}$ solution.

**Figure 6:**
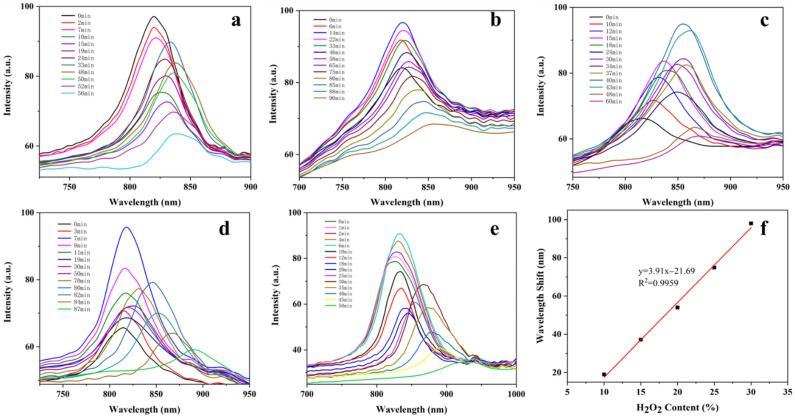
The maximum redshift of photonic crystal hydrogel with 15% BSP content in different concentrations (10% (**a**), 15% (**b**), 20% (**c**), 25% (**d**), 30% (**e**)). (**f**) The linear relationship between the maximum redshift of photonic crystal hydrogel and concentration of $H_{2}O_{2}$.

BSP has a good antioxidant effect based on the scavenging effect of BSP on hydroxyl radicals [43]. Hydrogen peroxide, $H_{2}O_{2}$, is a good donor of hydroxyl radicals [44]. Thus, we explored the responsiveness of BSP-PAM PhCs hydrogel to $H_{2}O_{2}$ solutions. We put BSP-PAM PhCs hydrogel with different BSP content (5%, 8%, 10%, 12%, 15%, and 20%) in 30% $H_{2}O_{2}$ solution, and under UV irradiation of 245 nm, the $H_{2}O_{2}$ solution produced hydroxyl radical. BSP acted as a hydrogen donor on hydroxyl radicals with the decomposition of 30%$\;H_{2}O_{2}$, which reduced the hydrogen bonding interaction between the BSP chain and PAM, weakened the cross-binding effect of BSP chain on PAM, and lead to further swelling of BSP-PAM PhCs hydrogel, resulting in a redshift of the reflection peak (Figure S3); PhCs hydrogel with 15% BSP content had the largest redshift (Figure 5). The BSP-PAM PhCs hydrogels with 15% BSP content showed different degrees of redshift with different concentrations of $H_{2}O_{2}$ (Figure 6a–e), and the maximum redshift (λmax) showed a good linear relationship with the concentration of $H_{2}O_{2}$ from 10% to 30%. (Figure 6f).

## 4. Conclusions

We constructed a BSP-based Semi-IPN network hydrogel by introducing BSP into the PAM hydrogel system, the macromolecular chains of BSP cross-wrap in the three-dimensional network of PAM through hydrogen bonding, and this biomass composite hydrogel not only maintains the excellent mechanical properties of a conventional PAM hydrogel but also obtains good biocompatibility. A novel biomass-based PhCs hydrogel was obtained by implanting a colloidal crystal array inside the hydrogel. The sensor is responsive to many organic solvents and vapors. Among them, the response to ethanol is the most obvious. It is not only sensitive to ethanol solutions, but also has good detection capability for ethanol vapor. In addition, based on the effect of BSP on hydroxyl radicals, the PhCs hydrogel is also responsive to $H_{2}O_{2}$ solution and has a significant linear relationship. Therefore, the obtained BSP-PAM PhCs hydrogel has excellent sensing performance and is expected to be used for the detection of the concentration of alcohol solutions and vapors, and the preliminary screening of the concentration of $H_{2}O_{2}$ solution.

## Data Availability

Not applicable.

## Supplementary Materials

The following supporting information can be downloaded at: https://www.mdpi.com/article/10.3390/bios12100841/s1, Figure S1: A photo of the *Bletilla striata*; Figure S2: SEM images of PMMA nanospheres (a), 3D photonic crystals (b), and particle size distribution of PMMA nanospheres (c); Figure S3: Response of PhCs hydrogels with different BSP contents (5% (a), 8% (b), 10% (c), 12% (d), 15% (e), 20% (f)) to 30% $H_{2}O_{2}$; Figure S4: Control group (**a**) response of PhCs hydrogels with 15% BSP content to $H_{2}O$. (**b**) response of PhCs hydrogels with 0% BSP content to $H_{2}O_{2}$.; Figure S5: Reflection wavelength–time relationship of PhCs hydrogels with 15% BSP content.
