# Supplementary material for: A Biomass Based Photonic Crystal Hydrogel Made of Bletilla striata Polysaccharide
Source: Biosensors (Basel). 2022 Oct 8;12(10):841. doi: 10.3390/bios12100841 (PMC9599764; doi:10.3390/bios12100841)
Supplement: Supplementary file 1 [file biosensors-12-00841-s001.zip › biosensors-1940391-supplementary.pdf]

biosensors

Supporting information

A Biomass Based Photonic Crystal Hydrogel Made of *Bletilla striata* Polysaccharide

Bo Sun, Wenxin Zhang, Yangyang Liu, Min Xue, Lili Qiu * and Zihui Meng *

School of Chemistry and Chemical Engineering, Beijing Institute of Technology, Beijing 100081, China
* Correspondence: qiulili@bit.edu.cn (L.Q.); mengzh@bit.edu.cn (Z.M.)

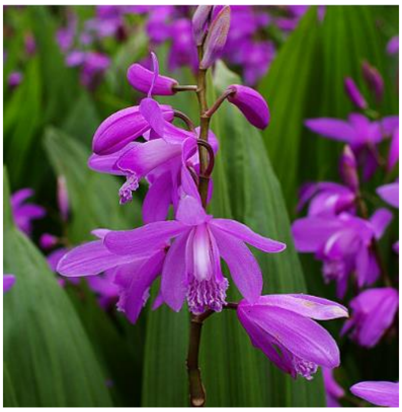

Figure S1. A photo of the *Bletilla striata*.

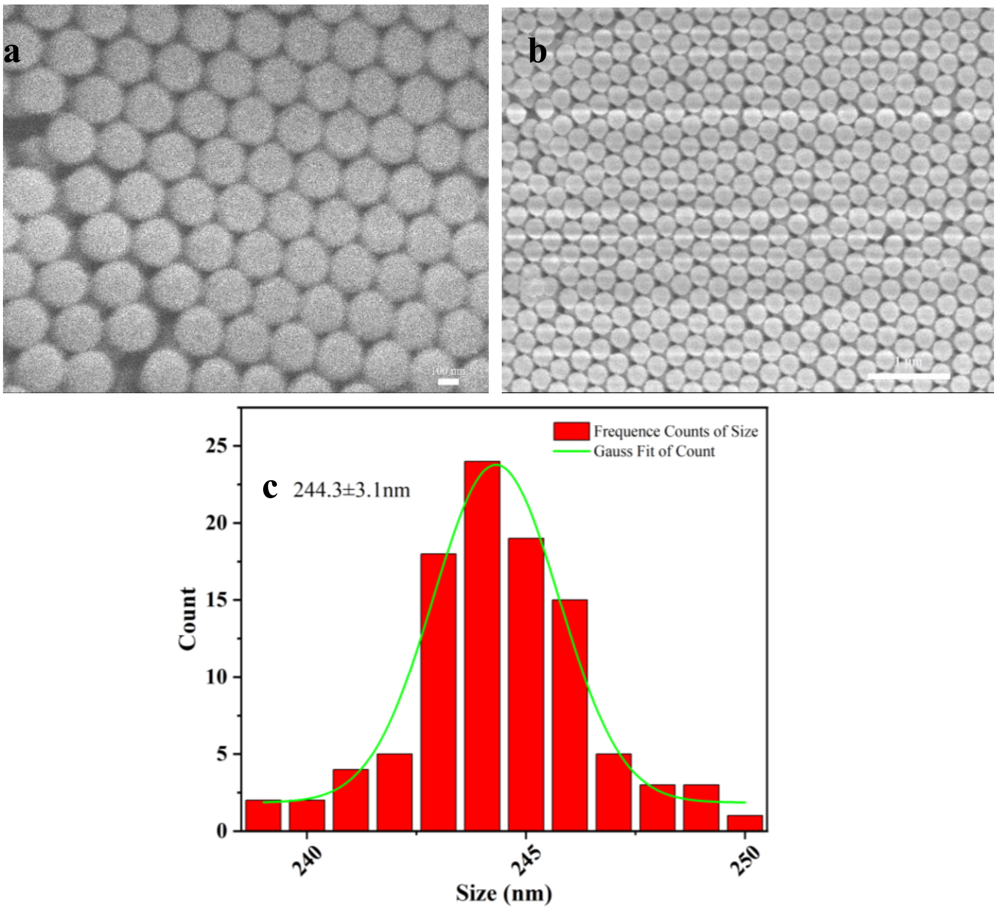

Figure S2. SEM images of PMMA nanospheres (**a**), 3D photonic crystals (**b**), and particle size distribution of PMMA nanospheres(**c**).

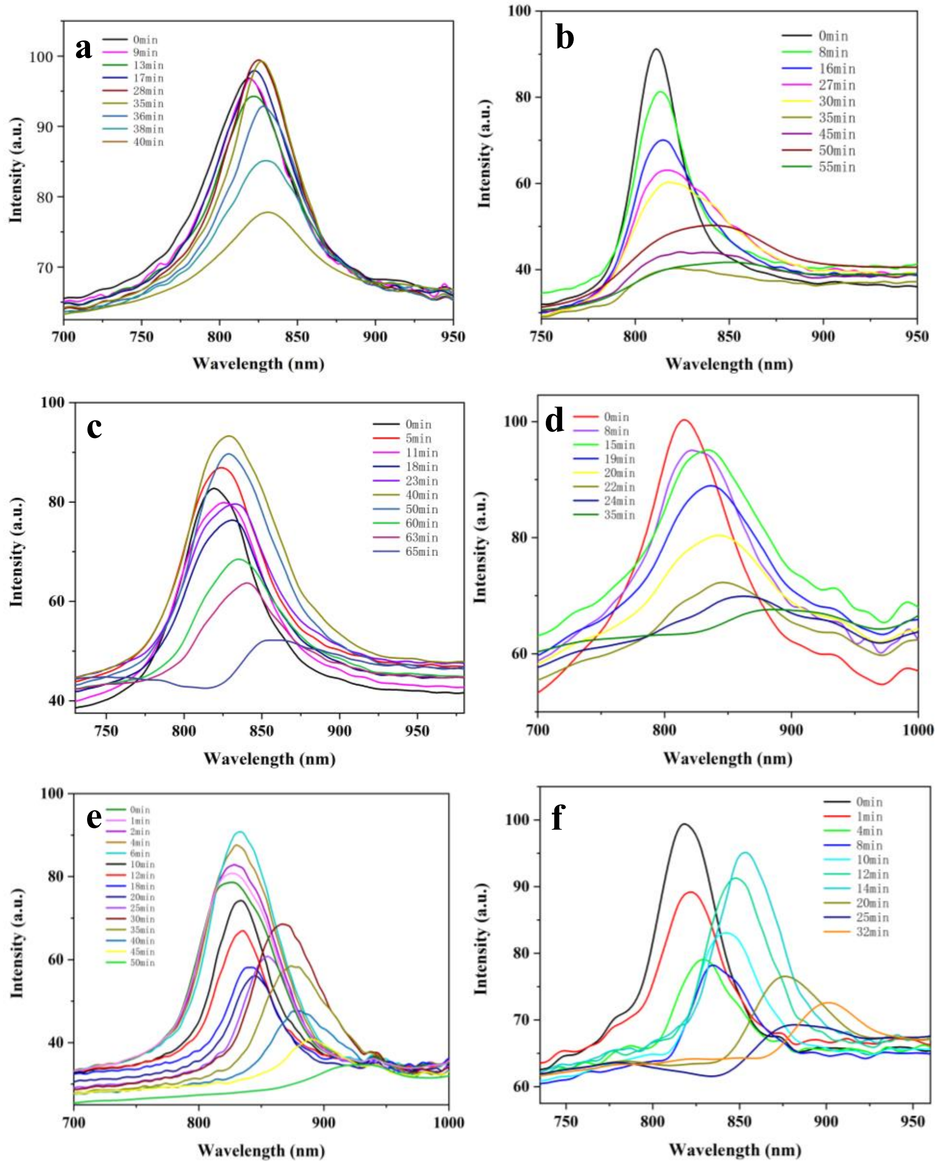

Figure S3. Response of PhCs hydrogels with different BSP contents(5% (**a**), 8% (**b**), 10% (**c**), 12% (**d**), 15% (**e**), 20% (**f**)) to 30% H_2O_2.

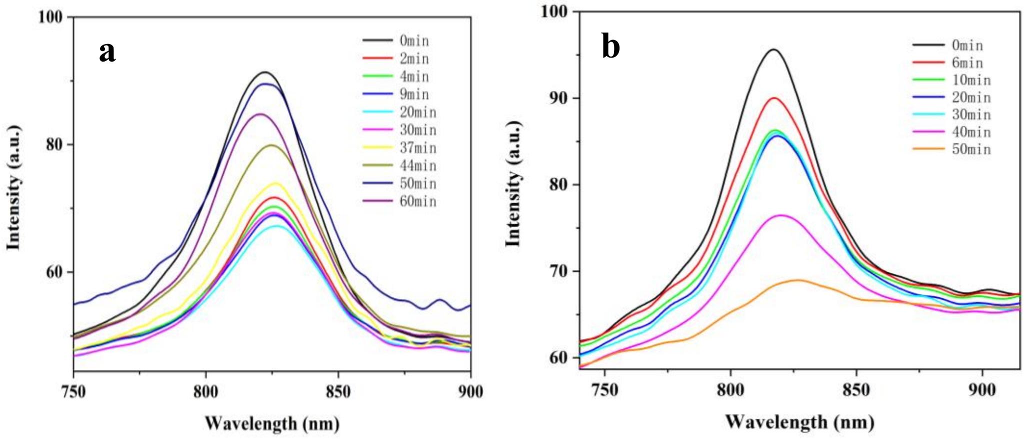

Figure S4. Control group (**a**) response of PhCs hydrogels with 15% BSP content to H_2O. (**b**) response of PhCs hydrogels with 0% BSP content to H_2O_2.

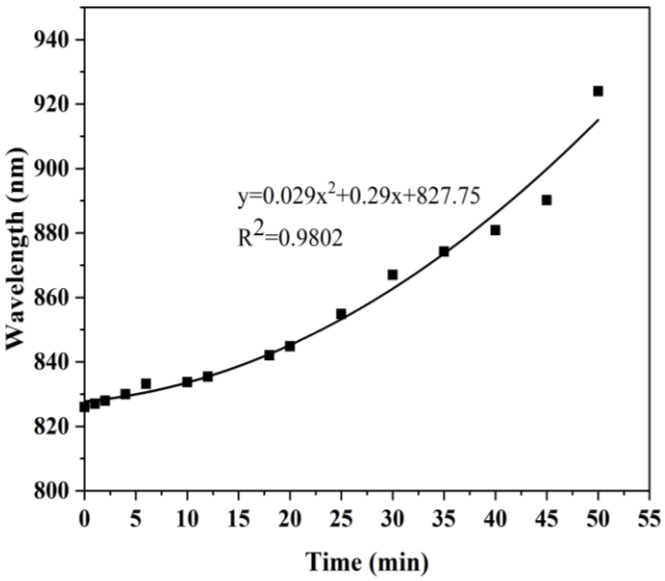

Figure S5. Reflection wavelength-time relationship of PhCs hydrogels with 15% BSP content.
